# Relationship between depression and quality of life among students: a systematic review and meta-analysis

**DOI:** 10.1038/s41598-023-33584-3

**Published:** 2023-04-25

**Authors:** Michele da Silva Valadão Fernandes, Carolina Rodrigues Mendonça, Thays Martins Vital da Silva, Priscilla Rayanne e Silva Noll, Luiz Carlos de Abreu, Matias Noll

**Affiliations:** 1 Instituto Federal de Educação, Ciência e Tecnologia Goiano, GO-154, Km 03, Ceres, Goiás 76300-000 Brazil; 2 Graduate Program in Health Sciences, School of Medicine, Universidade Federal de Goiás, Goiânia, Brazil; 3 Instituto Federal do Triângulo Mineiro, Uberaba, Minas Gerais Brazil; 4 Universidade de São Paulo, São Paulo, Brazil; 5 Universidade Federal do Espírito Santo, Vitória, Espirito Santo Brazil; 6 Universidade Federal de Goiás, Goiânia, Brazil; 7 Rede Estadual e Municipal de Educação de São Luís de Montes Belos, Ceres, Goiás Brazil

**Keywords:** Psychology, Diseases, Risk factors, Signs and symptoms

## Abstract

The objectives of this systematic review were to estimate the prevalence of depression and to identify the relationship between depression and quality of life (QOL) among high school and university students. Literature search was performed in the Scopus, Embase, PubMed, Scielo, CINAHL and Web of Science databases, following the PRISMA methodology. The results were presented through descriptive approaches and meta-analysis. Thirty-six studies met the eligibility criteria, and twenty-six were included in the meta-analysis. The prevalence of depressive symptoms was 27% (95% CI 0.21–0.33) among students, being high school and university students was 25% (95% CI 0.14–0.37) and 27% (95% CI 0.20–0.34), respectively, and most studies have shown that depression was associated with low QOL. Among the limitations of the study is the difficulty of generalizing the results found, considering the large sample of health students. New studies should be conducted considering the severity, duration, and patterns of depressive symptoms in high school and university students, to better understand the relationship between depression and QOL.

## Introduction

Depression is a disorder that increasingly affects different populations, with an estimated prevalence rate of 4.4% worldwide ^1^. This condition is defined as a mental disorder characterized by a persistent state of depressed mood, accompanied by other psychiatric symptoms such as fatigue and loss of energy, decreased interest or pleasure, impaired sleep, psychomotor agitation or retardation, concentration difficulties, change in appetite and weight, feelings of worthlessness or excessive guilt, or suicidal ideations ^2,3^. Biological, psychological, cultural, and social factors can contribute to the risk of depression at some stage of life ^4–7^. The high prevalence of depressive symptoms among high school and university students is a worrying aspect from the point of view of public health and educational policies ^8–12^, because it interferes negatively with learning, performance, and academic success ^13,14^, in addition to increasing the global burden of diseases ^3,15^.

High school and university students present significant risk factors for depression, since they need to deal with academic stress on a daily basis ^16–19^. This population is extremely concerned about school performance; emotional, family, and social conflicts; anxiety; among other aspects of life, common to adolescents and young adults, who need to adapt to changes in puberty ^18,20–22^. On the other hand, interaction with a supportive environment in the educational context can contribute to the prevention and remission of depressive symptoms, improving the QOL among students ^23,24^. Although different studies have shown that depression negatively impacts the QOL ^25–28^, the relationship between the severity of depressive symptoms and QOL among high school and university students is unclear ^21,29^.

Recent literature reviews have reported on the prevalence of depression in adolescents and their relationship with distinct biopsychosocial variables ^4,22,30^, such as academic stress, sociodemographic correlates ^12,31^, resilience ^32^, school frequency ^33^, and the school psychosocial climate ^34^. Other reviews, with samples of university students, also prioritized the results of depression prevalence ^35,36^ and a wide variety of associated risk factors, such as sleep quality ^37^, suicidal ideation ^36,38^, sex ^10,36,39^, socioeconomic status ^40^, and sexual abuse ^39^. No systematic reviews that analyzed the relationship between depression and QOL among high school and university students were found. The evaluation of QOL can contribute to preventive actions in the context of depression, since it is a multidimensional concept that covers well-being and satisfaction with different areas of life ^41–43^.

Assessing the relationship between depression and QOL is important for a broader understanding of the nature of diseases people are exposed to ^21,44,45^. Understanding how the different degrees of depression affect QOL and whether QOL interferes with the progression of the severity of depressive symptoms is necessary, since evidence shows that the trajectory of depressive symptoms vary within the same population ^46–48^. Thus, the objectives of this study are: (1) to estimate the prevalence of depression among high school and university students and (2) to identify the relationship between depression and QOL among high school and university students through a systematic review of the literature and a meta-analysis. In addition, we aimed to summarize the evidence of the influence of depression and QOL on academic performance, absenteeism, and school dropout rates among these students. The consolidation of these findings is essential to identify and clarify the risk factors for depression among adolescents and young people. In this way, it will be possible to guide future research and interventions focusing on improving students' mental health.

## Methods

### Research questions

The main research questions guiding this systematic review are, “What is the prevalence of depression among high school and university students?” and “What is the evidence on the relationship between depression and QOL among high school and university students?” The secondary question guiding this review is “What are the influences of depression and QOL on academic performance, absenteeism, and school dropout rates among high school and university students?” If the high prevalence of depression among high school and university students is related to self-perception of quality of life, it is possible that this relationship is determined by specific dimensions of QOL and manifests itself in different ways among students.

### Protocol and registration

The present systematic review was conducted according to the methodology for Preferred Reporting Items for Systematic Reviews and Meta-Analyses (PRISMA) ^49^, for identification, screening, eligibility, and inclusion of studies. Details that are more specific can be found in the registration of the International Prospective Register of Systematic Reviews and in the published protocol article ^50^. As the analysis was based on published articles (secondary data), ethical approval was not necessary.

This review follows the population, exposure, comparator, outcome (PECO) structure, mentioned in the recommended notification items for systematic reviews ^51^. Thus, “P” represents high school and university students; “E”, depression and QOL; “C”, sex and age group; and “O”, depression and QOL ^51^. Academic performance, absenteeism, and school dropout rates were also analyzed as secondary outcomes.

### Search strategy and eligibility criteria

In January 2023, a researcher (reviewer 1) accessed the Scopus, Embase, PubMed, Scielo, CINAHL, and Web of Science databases, restricting the search to publications in English between 2011 and 2023. The choice to limit the search to the last 13 years was guided by the following factors: (a) focusing on recent publications in the area, particularly those that assessed depression based on the current criteria of the Diagnostic and Statistical Manual of Mental disorders (DSM-5), published in 2013 ^52^ is more relevant, and (b) a prior analysis, based on PubMed, showed that publications and the production of research citations in this area were significantly increasing from 2011 onwards.

Table 1 shows the search strategy adapted to the different databases. The search strategy was also complemented by: (a) tracking of the references of the included studies and relevant systematic reviews, and (b) searches in Google Scholar. The main search keywords were: “high school students”, “college students” (population), “depression” (exposure/outcome) and “quality of life” (exposure/outcome).

**Table 1:** Search strategy.

#1	(depression OR “depressive symptoms” OR “depressive disorder” OR “depressed mood” OR “major depression” OR “mood disorder”)
#2	(adolescents OR teenagers OR adolescence OR teen OR youth OR young OR “young adult” OR “high school students” OR “secondary school students” OR students OR “college students” OR “university students”)
#3	(“quality of life” OR “health-related quality of life” OR wellbeing OR “personal satisfaction” OR HRQOL OR QOL OR “value of life”)
#4	#1 AND #2 AND #3

Depression was defined as any depressive disorder based on a clinical diagnosis, according to the criteria of the International Statistical Classification of Diseases and Related Health Problems ^53,54^ or the DSM ^52^, or by the evaluation of depressive symptoms through a validated inventory/self-reporting questionnaire ^55,56^. QOL was defined, according to the criteria of the World Health Organization (WHO), as “individuals’ perception of their position in life in the context of the culture and value systems in which they live and in relation to their goals, expectations, standards and concerns” ^57^.

Observational studies (cross-sectional and longitudinal) with the following characteristics were included: (a) a sample of high school and university students aged 10–33 years; (b) depression and QOL as the main outcome or exposure/risk factor; (c) reported the association between depression and QOL; (d) used a standardized questionnaire for QOL or health related QOL (HRQOL); and (e) evaluation of depression/depressive symptoms with validated instruments and/or clinical diagnosis. The age range 10 to 33 years was used based on the age of adolescents and young adults (age, 10 to 24 years as defined by WHO) ^57^. The age was extended to 33 years because the average age of university students is higher in recent years.

The exclusion criteria were: (a) theses, dissertations, books, book chapters, reviews, case reports, comments, letters and editorials, duplicate articles, and articles in which the full text could not be retrieved in online databases, through library requests, or by e-mails sent to the author(s) of the study; (b) studies with specific populations (pregnant and breastfeeding women, victims of violence, amputees, inpatients, and disabled people; in disaster situations, athletes, asthmatics, diabetics, and hypertensive people; patients with HIV, cancer, arthritis, cystic fibrosis, among other chronic diseases); (c) studies with samples of mixed ages, unless data could be collected, organized or calculated separately; (d) incomplete data on the association between depression and QOL; (e) clinical trials and case–control studies; and (f) when more than one article provided data on the same sample.

### Training of researchers

Before beginning the screening process, the researchers who participated in the eligibility assessments were subjected to training as to the inclusion/exclusion criteria of the study, with a practical session on eligibility assessment of 50 abstracts ^58^. In addition, the researchers participated in another training session to standardize the risk of bias and the analysis of Newcastle–Ottawa Scale (NOS), evaluating five articles not included in the present study. Finally, the researchers were trained on how to correctly use the Rayyan software and standardize the procedures ^58^.

### Review process

**Figure 1:**
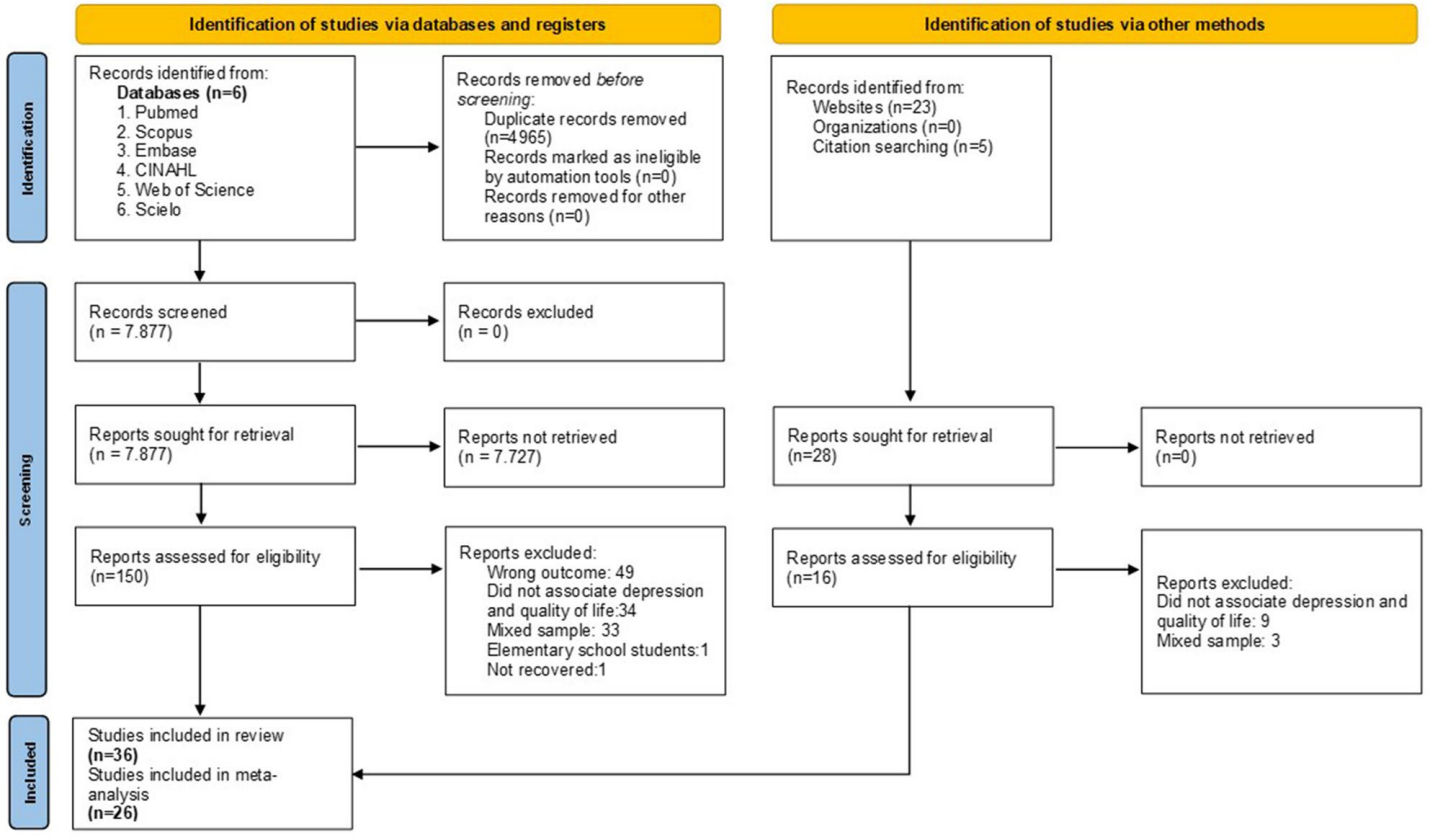
Flow diagram of the selection criteria for the study. Flowchart: Adapted from the PRISMA 2020 Flow Diagram.

After the bibliographic search, the articles retrieved in the databases were compared and the duplicates removed using EndNote X9 (Clarivate, PA, USA). In the first phase of the review, two researchers (reviewer 1 and reviewer 2) independently sorted the titles and summaries of all articles that met the inclusion and exclusion criteria. This phase was performed using Rayyan software (Rayyan Systems Inc., Cambridge, MA, USA) in blind mode ^59^. Disagreements regarding the inclusion and exclusion criteria were discussed and resolved by a third researcher (reviewer 3). In the second phase, the selected articles were fully read by two researchers (reviewer 1 and reviewer 4) and evaluated to determine their eligibility. The reliability between evaluators for the inclusion and exclusion of the studies was determined by calculating the percentage of concordance and the Cohen’s kappa coefficient ^58^. Finally, the eligible articles were included in the systematic review. The reference lists of the included articles were evaluated to identify possible additional studies lost in the database searches. Figure 1 shows the flowchart of this systematic review.

### Risk of bias and quality assessment of individual studies

The methodological quality and risk of bias among the studies were assessed by two researchers (reviewer 1 and reviewer 2) independently and with consensus. The methodological quality of the studies was evaluated using the online version of the Grading of Recommendations, Assessment, Development, and Evaluations (GRADE) tool ^60,61^. The strength of evidence of the studies was classified into four categories: high (four circles filled), moderate (three circles filled), low (two circles filled), or very low (one circle filled) ^60,61^. Factors such as the risk of bias, inconsistent results, indirect evidence, imprecision, and publication bias might decrease the quality of the evidence of the studies. However, the great magnitude of the effect, the dose–response gradient, and the presence of confounders in the reduction of the effect found are factors that could increase the quality of the evidence in the studies.

The NOS for observational studies ^62^ was used to assess the risk of bias. The adapted scale for cross-sectional (seven items) and cohort (eight items) studies consists of three dimensions that take into account the selection of participants, the comparability of the result groups, and the evaluation of the result measurements ^38^. All studies could receive a maximum of one star for each item, except for comparability, in which up to two stars could be assigned. The studies were considered as having a low risk of bias (≥ 3 points) or high risk of bias (< 3 points) ^38^. In addition, we assessed whether the authors provided a statement on conflicts of interest and information on ethical approval.

### Data extraction and evidence synthesis

The following information was collected from the studies using a standard data extraction spreadsheet: authors, year of publication, site/country, study design, follow-up period (longitudinal studies), characteristics of the participants (sample size, sex, and age range/mean age), instruments for the assessment of depression with respective cutoff points, QOL evaluation instruments, main findings, and association values.

Data regarding the prevalence of depression and association measures were collected, in addition to other additional results that refer to factors associated with depression and QOL. The results were categorized into two groups: (a) high school students and (b) university students. Data were collected and evaluated by two independent researchers (reviewer 1 and reviewer 4) and disagreements were resolved by a third researcher (reviewer 2).

The prevalence of depression and the results of the association between depression and QOL among students are presented as the main outcomes. The results of the prevalence of depression in the studies analyzed were presented according to the intensity of depressive symptoms. The different QOL domains evaluated were also considered in synthesizing the evidence. Secondary results are presented, including additional variables that are associated with students’ depression and QOL. We also described whether the studies presented results on the influence of depression and QOL on academic performance, absenteeism, and evasion. When possible, the differences between the sexes and age groups in terms of the prevalence of depression and the level of QOL among the students were compared.

### Meta-analysis

A meta-analysis was conducted using the random effects model with data on the prevalence of depression among high school students, depression among university students, and moderate and low QOL. The data are graphically displayed in Forest plots, showing prevalence rates with their 95% confidence intervals (CIs). Publication bias was evaluated using Egger’s test. All analyses were conducted using Stata version 16.0 (StataCorp LLC, College Station, TX, USA).

## Results

### Literature search and study selection

Figure 1 shows the selection process for this systematic review. In all, 12,842 articles were identified based on the eligibility criteria, and 28 additional articles were identified through lists of references and manual searches. After excluding duplicate articles, 7,877 articles were selected for title and abstract reading. There was moderate agreement (agreement = 99.4%, kappa = 0.60) between researchers and 150 articles remained for full text evaluation. After the full text analysis, 36 studies met the eligibility criteria and were included in the systematic review (Fig. 1). The articles included analyzed depression and QOL among high school and university students and provided information on the relationship between depression and QOL (Table 2)^44,63–97^.

**Table 2:** Detailed risk of bias results assessed using the Newcastle–Ottawa scale for assessing quality of observational studies.

References	Conflict of interests	Ethical approval	Newcastle–Ottawa scale for assessing observational studies										GRADE
			SC			CO		O					
			A	B	C	D	E	F	G	H	Total	Score^#^	
*Cross-sectional studies*													
Albani et al.^80^	Yes	Yes	1	0	0	1	0	1	1	–	4/8	50%	●●●●
Al-fayez and Ohaeri^92^	No	Yes	1	1	1	1	2	1	1	–	8/8	100%	●●○○
Assana et al.^74^	Yes	Yes	1	1	0	1	1	1	1	–	6/8	75%	●●●○
Alvi et al.^89^	Yes	*	1	0	0	1	0	1	1	–	4/8	50%	●●○○
Angkurawaranon et al.^64^	No	Yes	1	1	1	1	2	1	1	–	8/8	100%	●●●○
Armoon et al.^68^	No	Yes	0	0	0	1	1	1	1	–	4/8	50%	●●○○
Blebil et al.^83^	Yes	Yes	1	1	0	1	0	1	1	–	5/8	63%	●●●○
Borges et al.^79^	Yes	Yes	1	0	0	1	0	1	1	–	4/8	50%	●●○○
Cleofas^44^	No	Yes	0	0	0	1	0	1	1	–	3/8	38%	●●○○
Fernandes et al.^77^	Yes	Yes	1	1	0	1	2	1	1	–	7/8	88%	●●●○
Gan and Rue^67^	*	Yes	0	0	0	1	0	1	1	–	3/8	38%	●●○○
Ghassab-Abdollahi et al.^76^	Yes	Yes	1	1	0	1	0	1	1	–	5/8	63%	●●○○
Gómez-Delgado et al.^75^	Yes	Yes	1	1	0	1	0	1	1	–	5/8	63%	●●○○
Jenkins et al.^94^	Yes	Yes	0	0	0	1	1	1	1	–	4/8	50%	●●○○
Karuniawati et al.^84^	Yes	Yes	1	1	0	1	0	1	1	–	5/8	63%	●●○○
Li et al.^66^	Yes	Yes	1	1	1	1	2	1	1	–	8/8	100%	●●●●
Markovic et al.^73^	Yes	Yes	1	0	0	1	0	1	1	–	4/8	50%	●●○○
Miguel et al.^82^	Yes	Yes	1	1	0	1	2	1	1	–	7/8	88%	●●●●
Pagnin and Queiroz^96^	No	Yes	1	1	1	1	1	1	1	–	7/8	88%	●●●●
Pekmezovic et al.^97^	*	Yes	1	1	1	1	2	1	1	–	8/8	100%	●●●○
Pillay et al.^65^	No	Yes	0	1	0	1	1	1	1	–	5/8	63%	●●○○
Ra and Cho^72^	Yes	Yes	1	0	0	1	0	1	1	–	4/8	50%	●●○○
Racic et al.^85^	No	Yes	0	0	1	1	1	1	1	–	5/8	63%	●●○○
Ratnani et al.^63^	No	Yes	1	1	0	1	0	1	1	–	5/8	63%	●●●○
Singh et al.^69^	No	Yes	1	1	0	1	0	1	1	–	5/8	63%	●●●○
Shin et al.^91^	Yes	Yes	1	1	0	1	0	1	1	–	5/8	63%	●●●○
Solanki et al.^87^	Yes	Yes	1	0	0	1	0	1	1		4/8	50%	●●○○
Souza et al.^95^	*	Yes	1	1	1	1	1	1	1	–	7/8	88%	●●●○
Stheneur et al.^71^	*	*	1	0	0	1	0	1	1		4/8	50%	●●○○
Tejoyuwono et al.^81^	*	Yes	1	0	0	0	0	1	1		3/8	38%	●●○○
Tekin^70^	*	Yes	0	0	0	1	0	1	1		3/8	38%	●●○○
Wen et al.^86^	Yes	Yes	1	1	0	1	0	1	1		5/8	63%	●●●○
Yang et al.^90^	*	Yes	1	1	0	1	0	1	1		5/8	63%	●●●○
*Cohort studies*													
Aqeel et al.^88^	*	Yes	1	0	1	1	2	1	1	1	8/9	89%	●●●○
Burger et al.^78^	Yes	Yes	1	0	1	1	0	1	1	1	6/9	67%	●●○○
Moutinho et al.^93^	Yes	Yes	1	1	1	1	1	1	1	1	8/9	89%	●●●○

Newcastle–Ottawa scale for assessing quality of observational studies—Cross-sectional studies: (A) Representativeness of the sample; (B) Sample size; (C) Non-respondents; (D) Ascertainment of the exposure (risk factor); (E) Control for important factor or additional factor; (F) Assessment of the outcome; (G) Statistic test.

Newcastle–Ottawa for Assessing Quality for Observational Studies—Cohort studies: (A) Representativeness of the sample; (B) Selection of the non-exposed cohort; (C) Ascertainment of exposure; (D) Outcome of interest not present at start of study; (E) Control for important factor or additional factor; (F) Assessment of the outcome; (G) Follow-up long enough form outcomes to occur; (H) Adequacy of follow-up of cohorts.

SC: Selection Criteria; CO: Comparability; O: Outcome.

^#^Score reaches 100% with 8 and 9 points for cross-sectional and cohort studies, respectively.

*not reported; –, not applied.

GRADE, Grading of Recommendations, Assessment, Development and Evaluations; one filled circle, very low quality; two filled circles, low quality; three filled circles, moderate quality; four filled circles, high quality.

### Risk of bias and quality of the evidence

The NOS scale scores ranged from three to nine points. The classification of studies with lower scores ^44,67,70,81^ was related to unclear description of confounding factors, unadjusted results for confounders, and comparability between respondents and non-respondents characteristic. All studies reached scores ≥ 3 and were evaluated as having low risk of bias (Table 2).

The strength of the evidence classified using the GRADE methodology indicated that the studies had low (n = 19, 53%), moderate (n = 13, 36%), and high (n = 4, 11%) quality (Table 2). The low and moderate quality was justified by the inaccuracy of the results of observational studies, the reduced sample size, and the effect produced by these studies. Seven studies ^67,70,71,81,88,90,95,97^ did not clearly specify conflicts of interest, and two studies did not report whether ethical approval was obtained ^71,89^ (Table 2).

### Characteristics of the studies

Table 3 presents the characteristics of the studies included in the review, grouped into the following categories: year of publication, region, study design, students' study modality, sample size and types of assessment instruments for depressive symptoms/depression and QOL. This review included studies of students of 20 nationalities and a total sample of 24,704 people. Most studies were published between 2014 and 2020 (n = 20, 55.6%), mainly with the Asian population (n = 21, 58.3%), and university students (n = 27, 75%). With the exception of a single study, all studies included samples of both sexes. The study design mainly covered cross-sectional studies (n = 15, 93.8%), with only one longitudinal study ^93^. The sample size ranged from 40 participants ^88^ to 4,467 participants ^92^, 75.0% of whom were university students (Table 3). The mean age of high school students ranged from 13.2 (± 2.1)^70^ to 16.9 (± 1.2) years^92^, while the mean age of university students ranged from 19.0 (± 1.1)^63^ to 22.8 (± 3.0) years^63^. Most of the studies included a sample of medical students ^63–65,67,76,79,81,82,87,89,93,96^ nursing students ^80,95^, and health students ^68,73,78,85,88,94^. Only six studies included a large sample of university students ^44,66,69,84,86,97^. No study evaluated the possible influences of depression and QOL on academic performance, absenteeism, and school dropout.

**Table 3:** Quantitative characteristics of the articles included in the systematic review (n = 36).

Characteristics	Categories	Number of studies (%)
Year of publication	2021–2023	13 (36.1)
	2017–2020	15 (41.7)
	2014–2016	5 (13.9)
	2011–2013	3 (8.3)
Region	Asia	21 (58.3)
	America	8 (22.2)
	Europe	6 (16.7)
	Africa	1 (2.8)
	Antarctica	0 (0.0)
	Oceania	0 (0.0)
Study design	Cross-sectional	33 (91.7)
	Longitudinal	3 (8.3)
Sex	Both sexes	35 (97.2)
	Male sex only	0 (0.0)
	Female sex only	1 (2.8)
Students	University students	27 (75.0)
	High school students	9 (25.0)
University students (n = 27)	Medical students	12 (44.4)
	Other courses	6 (22.2)
	Health students	7 (26.0)
	Nursing students	2 (7.4)
High school students (n = 9)	Academic formation	7 (87.5)
	Vocational-technical school	1 (12.5)
Sample size	< 300	17 (47.2)
	300–1000	11 (30.6)
	1000–2000	5 (13.9)
	> 2000	3 (8.3)
Assessment of depression	BDI	9 (25.0)
	DASS-21	6 (16.7)
	PHQ	5 (13.9)
	CDI	3 (8.3)
	CES-D	3 (8.3)
	HADS	3 (8.3)
	ZUNG SDS	2 (5.5)
	MHI-38	1 (2.8)
	TSCC	1 (2.8)
	ADRS	1 (2.8)
	RCADS-P	1 (2.8)
	SCL-90-R	1 (2.8)
Assessment of QOL/HRQOL	WHOQOL	19 (52.8)
	SF-36	7 (19.4)
	KIDSCREEN	2 (5.5)
	PedsQL	2 (5.5)
	EQ-5D	1 (2.8)
	YQOL-SF	1 (2.8)
	VERAS-Q	1 (2.8)
	SF-12	1 (2.8)
	COV19-QOL	1 (2.8)
	OK-ados	1 (2.8)

ADRS: Adolescent Depression Rating Scale; CES-D: Center for Epidemiological Studies Depression Scale; DASS: Depression, Anxiety, and Stress Scale; DS: Domain scores; EQ-5DVAS: Visual Analogue Scale; ES: Effect size; HADS: Hospital Anxiety and Depression scale; HRQOL: health-related quality of life; CI: confidence interval; MHI-38—Mental health inventory; NR: not reported; OK-ados: OK-ados questionnaire; OR: odds ratio; PedsQL: Pediatric Quality of life Inventory; PHQ: Patient Health Questionnaire; QOL: quality of life; RAND 36-Item Health Survey; RCADS-P: Revised children anxiety and depression scales, parent form; SF-8: Optum Short Form-8 Health Survey; SF-36: Short Form Health Survey; TSCC: Trauma Symptom Checklist for Children; WHOQOL: The World Health Organization Quality of Life Questionnaire; WHOQOL-BREF: The World Health Organization Quality of Life Questionnaire—short version; WHOQOL-BREF-THAI: The World Health Organization Quality of Life Questionnaire—Thai version; YQOL-SF: Life Instrument-Short Form; ZUNG SDS: Zung self-rating depression scale.

### Characteristics of results and main findings

The characteristics and main results are presented separately for the evaluation of depression and QOL among students, prevalence of depression and its relationship with QOL among students, other factors associated with depression and QOL among students, and meta-analysis.

#### Evaluation of depression and quality of life among students

Table 3 shows a summary of the instruments used to assess depressive symptoms and Table 4 lists the respective cutoff points adopted in each study. The most widely used instrument for assessing depression and depressive symptoms was the Beck Depression Inventory (BDI) (n = 9, 25.0%), with cutoff points ranging from ≥ 10 to > 15 for the presence of depressive symptoms. Other studies used a variety of instruments to assess depression and depressive symptoms, including the Depression Anxiety Stress Scale (DASS-21) (n = 6, 16.7%) and the Zung Self-Rating Depression Scale (ZUNG SDS) (n = 2, 5.5%) ^65,85^.

**Table 4:** Characteristics of the studies included and their outcome variables.

References	Country	Study design	Sample size/% female participants	Age MD (SD)	QOL Assessment/Domain Scores	Depression assessment/Cutoff point	Prevalence of depression/MD (SD) Depression Scores
*High school students*							
Al-fayez and Ohaeri^92^	Kuwait	Cross-sectional	n = 4.467 (51.4%)	16.9 ± 1.2	WHOQOL-BREF DS: Physical Psychological Social relations Environment	TSCC	Female: 13.9 ± 3.9 Male: 12.9 ± 3.8
Assana et al.^74^	Thailand	Cross-sectional	n = 1.112 (50%)	16.4 ± 0.94	WHOQOL-BREF-THAI DS: Physical Psychological Social relations Environment	CES-D Cutoff point* > 22	n = 415 (37%)
Fernandes et al.^77^	Brazil	Cross-sectional	n = 343 (55.7%)	16.1 ± 0.93	WHOQOL-BREF	CDI Cutoff point* > 17	n = 143 (43.4%)
Gómez-Delgado et al.^75^	Mexico	Cross-sectional	n = 1. 446 (64.9%)	16.1 ± 0.8	KIDSCREEN-52 DS: Physical well-being Psychological well-being Mood and emotions self-perception Autonomy Parent relations and home life Financial resources Social Support School environment Social acceptance	CDI Cutoff point* > 19	n = 319 (21.2%)
Ra and Cho^72^	Republic of Korea	Cross-sectional	n = 385 (55.7%)	13.9 ± 0.54	KIDSCREEN-10 DS: Physical activities and health General mood and feeling about themselves Family and free time Friends School Learning during the previous week	CDI Cutoff point* > 19	n = 69 (17.9%)
Shin et al.^91^	Republic of Korea	Cross-sectional	n = 291 (100%)	16.4 ± 1.5	PedsQL DS: Physical Emotional Social School functioning	CES-D	34.7 ± 9.0
Stheneur et al.^71^	France	Cross-sectional	n = 855 (47.2%)	16.6 ± 0.9	OK-ados DS: Recreation and relationships with others School Family and adult life Esteem and self-image	ADRS Cutoff point* ≥ 6	n = 73 (8.5%)
Tekin^70^	Turkey	Cross-sectional	n = 118 (65.0%)	13.2 ± 2.1	PedsQL	RCADS-P	Pre-pandemic COVID-19: 52.2 ± 12.2 Pandemic COVID-19: 58.5 ± 14.4
Yang et al.^90^	China	Cross-sectional	n = 1.402 (63.3%)	16.5 ± 1.9	SF-36	SCL-90-R Cutoff point* ≥ 2.5	Grade 1: 1.74 ± 0.69 Grade 2: 1.70 ± 0.65 Grade 3: 1.82 ± 0.74
*University students*							
Albani et al.^80^	US	Cross-sectional	n = 200 (86.5%)	22.8 ± 12.2	SF-36 DS: Physical component Mental component	HADS Cutoff point* ≥ 8	n = 63 (31.5%)
Alvi et al.^89^	Pakistan	Cross-sectional	n = 200 (44.0%)	21.5 ± 2.4	WHOQOL-BREF	DASS-21	6.62 ± 4.51
Angkurawaranon et al.^64^	Thailand	Cross-sectional	n = 1.014 (53.1%)	20.8 ± 1.5	SF- 36	PHQ-9	n = 100 (10%) Mild = 8.4% Moderate/severe = 1.5%
Aqeel et al.^88^	Pakistan	Longitudinal Follow-up: 5 Month T0—1 Month into Lockdown T1—3 weeks into Lockdown T2—4 months into Lockdown	n = 40 NR	21.6 ± 1.1	WHOQOL-BREF	BDI Cutoff point* ≥ 14	n = 16 (40.0%)
Armoon et al.^68^	Iran	Cross-sectional	n = 275 (68%)	22.1 ± 3.6	WHOQOL-BREF	DASS-21	n = 20 (7%)
Blebil et al.^83^	Malaysia	Cross-sectional	n = 371 (77.6%)	21.2 ± 1.5	WHOQOL-BREF	PHQ-9 Cutoff point* ≥ 10	n = 176 (61.1%) Mild depression = 42.1% Moderate depression = 17.4% Severe depression = 1.9%
Borges et al.^79^	Brazil	Cross-sectional	n = 139 (53.2%)	21.1 ± 2.6 to 25.1 ± 2	WHOQOL-BREF	HADS	NR
Burger et al.^78^	Germany	Longitudinal Follow-up: 5 semesters	n = 163 (68.7%)	18–32 years	SF-12 DS: Physical component Mental component	BDI-II Cutoff point* ≥ 14	n = 32 (19.6%)
Cleofas^44^	Philippines	Cross-sectional	n = 249 (89.6%)	20–22 years	YQOL-SF DS: NR	MHI-38	12.6
Gan and Rue^67^	Malaysia	Cross-sectional	n = 149 (57%)	22–24 years	WHOQOL-BREF	HADS Cutoff point* ≥ 8	n = 17 (11%) Borderline/mild symptoms = 8% Significant symptoms = 3.4%
Ghassab-Abdollahi et al.^76^	Iran	Cross-sectional	n = 186 (50.0%)	22.6 ± 2.8	WHOQOL-BREF	BDI-II	n = 7 (3.8%)
Jenkins et al.^94^	England	Cross-sectional	n = 285 (86.8%)	20.51 ± 4.19	SF-36	PHQ-2 Cutoff point* ≥ 3	n = 98 (34%)
Karuniawati et al.^84^	Indonesia	Cross-sectional	n = 606 (81.0%)	17–27 years	WHOQOL-BREF	DASS-21 Cutoff point* > 9	n = 351 (57.9%) Mild depression = 18.5% Moderate depression = 24.4% Severe depression = 8.3% Very severe = 6.8%
Li et al.^66^	China	Cross-sectional	n = 2.312 (74.4%)	20.3 ± 1.6	WHOQOL-BREF	BDI-II Cutoff point* ≥ 14	n = 668 (29%)
Markovic et al.^73^	Serbia	Cross-sectional	n = 797 (74%)	21.7 ± 2.4	COV19-QOL DS: Quality of life Mental health Physical health Anxiety Depression Personal safety	PHQ-9 Cutoff point* ≥ 10	n = 248 (31.2%)
Miguel et al.^82^	Brazil	Cross-sectional	n = 1.305 (52.9%)	22.8 ± 3.01	VERAS-Q DS: Time management Psychological health Physical health Learning environment	BDI	6.02 (95% CI 5.90, 6.13)
Moutinho et al.^93^	Brazil	Longitudinal Follow-up: 2 years	n = 312 (64.1%)	21.0 ± 26	WHOQOL-BREF	DASS-21	n = 93 (30%)
Pagnin and Queiroz^96^	Brazil	Cross-sectional	n = 193 (53.9%)	21.42 ± 2.41	WHOQOL-BREF	BDI Cutoff point* ≥ 10	n = 117 (61%) Mild–Moderate = 31.6% Moderate/severe = 22.3% Severe = 13%
Pekmezovic et al.^97^	Republic of Serbia	Cross-sectional	n = 1.624 (53.7%)	20.8 ± 1.8	SF-36	BDI Cutoff point* ≥ 11	n = 357 (22%) Mild depression = 15.1% Moderate depression = 4.1% Severe depression = 2.8%
Pillay et al.^65^	South Africa	Cross-sectional	n = 230 (71.3%)	21 (18–32) years	WHOQOL	ZUNG SDS Cutoff point* ≥ 30	n = 166 (72%) Moderate symptoms = 60.9% Severe symptoms = 15.6%
Racic et al.^85^	Bosnia and Herzegovina and Republic of Serbia	Cross-sectional	n = 426 (69.2%)	21.5 ± 2.26	EQ-5D VAS DS: NR	ZUNG SDS Cutoff point* ≥ 45	n = 16 (4%)
Ratnani et al.^63^	India	Cross-sectional	(n = 290) 53%	19 ± 1.1	WHOQOL-BREF	BDI Cutoff point* > 13	n = 26 (9%)
Singh et al.^69^	India	Cross-sectional	n = 150 (64%)	18–22 years	WHOQOL-BREF	DASS-21	Medical students: 6.0 ± 5.3 Engineering students: 3.88 ± 3.11 Arts students: 3.44 ± 2.71
Solanki et al.^87^	India	Cross-sectional	n = 395 (61.0%)	20.9 ± 1.9	WHOQOL-BREF	CES-D Cutoff point* > 16	n = 145 (36.7%)
Souza et al.^95^	Brazil	Cross-sectional	n = 256 (80.5%)	21.5 ± 2.9	SF-36	BDI Cutoff point* > 15	n = 36 (14%)
Tejoyuwono et al.^81^	Indonesia	Cross-sectional	n = 361 (74.2%)	18–32 years	WHOQOL-BREF	DASS-21 Cutoff point* > 9	n = 12 (3.3%) Mild depression = 2.2% Moderate depression = 0.8% Severe depression = 0.3%
Wen et al.^86^	China	Cross-sectional	n = 2.757 (58.5%)	19.07 ± 1.14	SF-36	PHQ-2 Cutoff point* ≥ 3	1.00 (95% CI 0.00, 2.00)

ADRS: Adolescent Depression Rating Scale; CES-D: Center for Epidemiological Studies Depression Scale; DASS: Depression, Anxiety, and Stress Scale; DS: Domain scores; EQ-5DVAS: Visual Analogue Scale; ES: Effect size; HADS: Hospital Anxiety and Depression scale; HRQOL: health-related quality of life; CI: confidence interval; MHI-38—Mental health inventory; NR: not reported; OK-ados: OK-ados questionnaire; OR: odds ratio; PedsQL: Pediatric Quality of life Inventory; PHQ: Patient Health Questionnaire; QOL: quality of life; RAND 36-Item Health Survey; RCADS-P: Revised children anxiety and depression scales, parent form; SF-8: Optum Short Form-8 Health Survey; SF-36: Short Form Health Survey; TSCC: Trauma Symptom Checklist for Children; WHOQOL: The World Health Organization Quality of Life Questionnaire; WHOQOL-BREF: The World Health Organization Quality of Life Questionnaire—short version; WHOQOL-BREF-THAI: The World Health Organization Quality of Life Questionnaire—Thai version; YQOL-SF: Life Instrument-Short Form; ZUNG SDS: Zung self-rating depression scale.

T, reference for the data collection period in the longitudinal study.

*, reference value for the presence of depressive symptoms.

Twelve studies did not specify the cutoff points adopted for the evaluation of depressive symptoms ^44,68–70,76,79,82,89,91–93,97^. There were no studies based on the clinical diagnosis of depression, and the evaluation of depressive symptoms is prevalent through self-reporting questionnaires. The severity of depressive symptoms was evaluated only in eight studies ^64,65,67,83,84,90,96,97^, in which the prevalence of depressive symptoms was categorized into mild, moderate, and severe/significant symptoms.

For the QOL evaluation, the most widely used instrument was the World Health Organization QOL Questionnaire (WHOQOL; WHOQOL-BREF) (n = 19, 52.8%), followed by the RAND 36-item Short Form Survey (SF-36) (n = 7, 19.4%), as specified in Table 3. The different QOL domains evaluated by the main instruments covered the physical, environmental, psychological, and social domains (WHOQOL; WHOQOL-BREF, and SF-36), and the sub-domains related to functional capacity, general health perceptions, bodily pain, vitality, social, physical, and mental functioning, and limitations caused by emotional problems (SF-36). Although there was a certain tendency for studies to assess QOL from different domains, ten studies did not analyze these domains/sub-domains ^44,66,68,71,72,74,78,85,88,89^.

#### Prevalence of depression and its relation to students’ quality of life

Table 4 shows a summary of the results on the prevalence of depression and its relationship with the students’ QOL, categorized by high school and university students, by the intensity of depressive symptoms and by instruments used in the evaluation of depression and QOL. The prevalence of depressive symptoms among high school students ranged from 8.5% among French students^71^ to 43.4% among Brazilian students^77^. Among college students, the prevalence of depressive symptoms ranged from 3.3% among Indonesian students^81^ to 61% among Malaysian and Brazilian students^83,96^. Table 5 shows the main results on the relationship between depression and QoL. Association/correlation tests for each study can be found in Supplementary File 1.

**Table 5:** Relation with depression and QV.

Main results	Study
*High school students (n = 9)*	
QOL is negatively correlated with depression	Al-fayez and Ohaeri^92^
	Assana et al.^74^
	Fernandes et al.^77^
	Ra and Cho^72^
	Shin et al.^91^
	Stheneur et al.^71^
	Tekin^70^
	Yang et al.^90^
HRQOL was significantly correlated with depression, except in the dimensions financial resources and social support	Gómez-Delgado et al.^75^
*University Students (n = 27)*	
Depression was predictive of QOL	Albani et al.^80^
	Ghassab-Abdollahi et al.^76^
	Miguel et al.^82^
QOL was predictive of depression	Alvi et al.^89^
Depression is associated with low QoL/HRQL	Angkurawaranon et al.^64^
	Aqeel et al.^88^
	Karuniawati et al.^84^
	Blebil et al.^83^
	Cleofas^44^
	Li et al.^66^
	Pagnin and Queiroz^96^
	Pillay et al.^65^
	Ratnani et al.^63^
	Solanki et al.^87^
	Tejoyuwono et al.^81^
QOL is negatively correlated with depression	Markovic et al.^73^
	Pekmezovic et al.^97^
	Wen et al.^86^
Depression is associated with QoL in the psychological domain	Borges et al.^79^
	Burger et al.^78^
Depressive symptoms are associated with lower general QOL, except with the general health domain and social domain	Gan and Rue^67^
Depression is not correlated with physical functioning and pain	Jenkins et al.^94^
Depression negatively impacts the physical and social domains	Singh et al.^69^
Moderate symptoms of depression negatively affect the mental and physical components	Souza et al.^95^
Depression is not correlated with physical functioning	Moutinho et al.^93^
Depression is not correlated with QOL	Armoon et al.^68^
	Racic et al.^85^

Studies with a sample of high school students identified that QoL is negatively correlated with depression (n = 8, 100%). Only one study showed that, regarding the QoL domains, the financial resources and social support dimensions were not correlated with depression among students from Mexico^75^. In general, studies with a sample of university students found that depression is associated with low QoL (n = 11, 40.7%). In addition, depression was a predictor of QoL and vice versa. On the other hand, other studies (n = 6, 22.2%) present a varied behavior regarding the relationship between different QOL domains and the prevalence of depressive symptoms. In Thai and Malaysian students, for example, depression was associated only with the psychological and physical domains of QOL ^64,67^, while a study with a sample of 193 Brazilian students indicated that the physical domain of QOL was unaffected by depression ^96^. In two studies depression is not correlated with QOL^68,85^.

Three studies analyzed the relationship between depressive symptoms and QOL among German, Brazilian and Pakistani students with a longitudinal design ^78,88,93^. The German students showed an increase in depression symptoms over the semesters, with highly significant correlations between depression and mental quality of life^78^. The presence of depressive symptoms among Brazilian students was negatively related to QOL in all domains, except for the physical domain ^93^. It also showed that students with depression at the beginning of graduation tend to maintain depressive symptoms over time, contributing to a worse future QOL ^93^. Female students were more likely to have a worse physical QOL over time ^93^. On the other hand, students with depression showed improvement in QoL during the COVID-19 epidemic lockdown in Pakistan^88^.

#### Other factors associated with depression and quality of life among students

In addition to the main results of interest, the studies presented other important variables that are associated with depression and QOL among students, such as anxiety and academic stress. According to one study, self-esteem was positively correlated with QOL, while anxiety symptoms, and relationship with their parents were negatively correlated with QOL in high school students ^92^. Another study analyzed that QOL was also correlated with low and moderate anxiety, with a high level of general well-being and with low/moderate level of educational stress ^74^.

Studies have shown that among university students, QOL was negatively correlated with anxiety ^44,67,94^ and emotional control ^44^, and positively correlated with general positive affection, emotional bonds, life satisfaction ^44^, and family income ^97^. Students who engaged in physical activity every day had higher scores on the HRQOL ^97^.

The frequency of depressive symptoms increased with increased anxiety ^63,85^, academic stress, sleep disorders, academic pressure ^66^, and perceived stress ^85^. Students with depression had higher scores for social phobia ^63^ and the intensity of depressive symptoms was higher in the last year of their undergraduate course ^95^. In a sample of Chinese students, depression was more prevalent among medical students, followed by engineering and arts students ^69^.

Seven studies evaluated depression and QOL of students during the COVID-19 pandemic^70,73,75,77,81,84,88^. In the pandemic period, the prevalence of depression ranged from 21.2% among Mexican high school students^75^ to 57.9% among Indonesian university students^84^. It was observed that the COVID-19 pandemic negatively affected the mental health and QOL of students^73,88^ and that depression symptoms were associated with poor quality of life and social isolation^70,75,77,81,88^.

#### Meta-analysis

**Figure 2:**
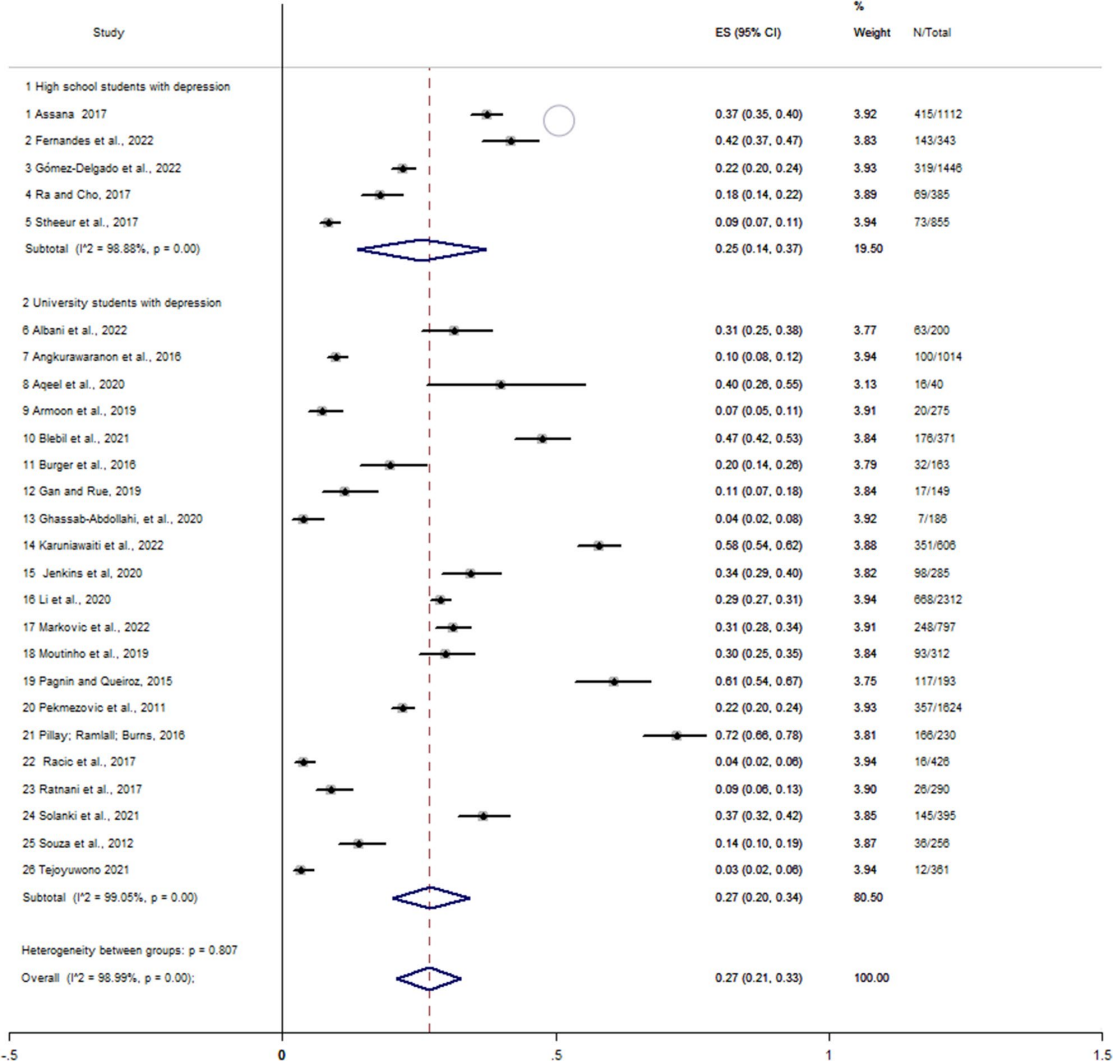
Forest plot evaluating the prevalence of depression in students, using data from 26 studies. Flowchart: Elaborated by the authors.

Figure 2 shows the combined prevalence of depression among high school students and depression among university students. The combined prevalence of depression among students was 27% (95% CI 0.21–0.33). The prevalence of depression among High school students was 25% (95% CI 0.14–0.37). The prevalence of depression among university students was 27% (95% CI 0.20–0.34).

There was a high level of statistical heterogeneity (*I*^2^ = 99.40%, *p* < 0.001). Heterogeneity had an influence on the result of the analysis. Evidence of publication bias in the meta-analysis of the combined prevalence was found using the Egger’s regression test (*p* = 0.000).

In the meta-analysis, involving three studies, the odds ratio for the association between depression and quality of life in students was 0.009 (95% CI − 0.009 to 0.027), (*I*^2^ = 95.6%, *p* < 0.01), not indicating a positive association^68,74,85^.

## Discussion

The present study systematically estimated the prevalence of depression and summarized the relationship between depression and QOL among high school and university students. The prevalence of depressive symptoms was 27% among students and most studies have shown that depressive symptoms was associated with a low QOL. Despite being relevant to research involving students, the studies did not evaluate the influence of depression and QOL on academic performance, absenteeism, and school dropout rates.

The main results show that the estimated prevalence rate of depression among university students was 27%, similar to the results of other meta-analyses that present the prevalence of depressive symptoms of 24.4% to 34.0% with the same population ^11,35,36,38,40^. About 25% of high school students had depressive symptoms. Indonesian and Brazilian high school students had a higher prevalence of depressive symptoms compared to students from Mexico, Republic of Korea and France. Differences in the prevalence of depression can also be observed in different studies, where the prevalence of depression was in Chinese, 24.3% ^12^, Pakistani (17.2%), and Malaysian (26.2%) students ^98,99^. However, high school students in Indonesia had a higher prevalence of depressive symptoms, with rates of 52.7% ^100^.

The findings of this review also demonstrate that high school and university students present a higher prevalence of depressive symptoms compared to large samples in distinct communities, ranging from 7.3% in countries like Australia to 20.6% in South American countries ^101^. Estimates of a 12-month depression prevalence in adolescents and young adults in the United States range from 8.7% to 11.3% ^102^, rates lower compared to those found in the present review.

The manifestations of depressive symptoms are not static, and they affect a distinct population of students ^45,93^, since there are several biological, psychological, and social factors that contribute to the risk of depression, including cultural determinants that are present in the person’s life such as the context of development, parental practices, and temperament ^48,98^. Part of the challenge relates to the heterogeneous nature of the diagnosis and condition of depression. There is an emerging notion that mood disorders lie on a spectrum ^103^. In addition, individuals of different ethnicities may express depression differently. Chinese, for example, tend to deny mental health symptoms or express them somatically ^104^. Given the complexity of identifying protection mechanisms and risk factors, research suggests that the dimensions of subjective well-being are complementary aspects of the evaluation of depression symptoms ^25,105,106^. In addition, QOL is an important indicator for identifying groups vulnerable to depressive symptoms and the golden objective for treating depression is to improve QOL ^21^.

In this review, 97.2% of the studies showed some type of association between depression and QOL, indicating that students with depressive symptoms tend to have worse QOL, or that QOL is a predictor of depression. The role of depressive symptoms as a negative predictor of QOL was documented in other reviews with adolescents ^9^ and university students ^107^. However, the main relevance of the present study is the fact that depressive symptoms may not impact in the same way in the different domains of QOL ^64,67,93,96^. The psychological dimension of the QOL of students seems to be the most affected; however, it is not possible to state precisely that it does not occur with the physical, environmental, and social dimensions of the QOL. This is because other factors associated with depression and QOL must be considered, such as the presence of chronic or physical diseases, for example ^108^.

Data from the meta-analysis indicate that there is no positive association between depression and QOL in students, showing a possible influence of other mediators on the relationship between depression and QOL. Some people, despite experiencing depressive symptoms at some stage of life, may present adaptive mechanisms that allow them to self-manage mental suffering and demonstrate resilience ^32,43,98,109–112^. The influence of different degrees of depressive symptoms may also compromise the analysis of results, but studies do not provide enough data to support this statement. Therefore, these findings are limited in clarifying the wide and complex relationship between depression and QOL among students. Further studies are needed, mainly with longitudinal design and with quality evidence.

With regard to QOL, the perception of QOL can be more positive or negative as for the meanings each person attributes to their life experiences ^111,113–116^ To better understand these aspects, the evaluation of QOL should consider the relationship between positive and negative psychological dimensions as independent but at the same time inter-related dimensions ^25^. In this sense, a favorable educational environment may play a “barrier” role in negative psychological dimensions among students, such as stress ^25^. The psychological, physical, environmental, and social domains of QOL present important differences when analyzed in terms of sex and geographic region ^64,93,95^. Female students tend to present worse QOL, in addition to having the most impaired physical domain of QOL ^93,96,117^, a condition that may be associated with the probability of women exercising less than men ^118^. This can also be explained by the fact that different instruments are used in the evaluation of QOL and by adverse cultural or social factors.

This study also showed that students experienced intense depressive symptoms and worsened QOL during the COVID-19 pandemic. Since the establishment of social distancing/isolation measures due to the COVID-19 pandemic caused by the SARS-CoV-2 virus, students have shown considerable increases in depressive symptoms and anxiety ^119,120^. In part, this is due to prolonged social isolation, bereavement, violence in the family context, and excessive use of the internet and social networks ^121–126^. The existence of social distancing implemented to prevent the spread of the COVID-19 virus caused limitations in physical and social activities, including leisure activities and in the sufficiency of the family's financial ^127^. In addition, the blockade and closure of schools and universities forced students to study at home, which may have contributed to increased symptoms of depression and consequent worsening of QOL^127,128^.

This review had some limitations. First, the assessment of depression and QOL in the studies considered different instruments, which made comparison of results difficult. Second, the most widely used instrument for the evaluation of depressive symptoms, the BDI, presented different cutoff points in the selected studies, which may reflect probable bias. In addition, screening tools are criticized for having a greater chance of false-positive results, making the burden of the disease seem worse ^129^. Depressive symptoms were measured using psychometric tools that indicated the presence or absence of symptoms, but they were not able to diagnose depression. A clinical evaluation would be essential to better understand and standardize the results ^21,42^. Third, most studies used a cross-sectional design, which does not allow definitive conclusions on causality. Longitudinal studies could demonstrate whether poor QOL is a predictor of depression or whether depression is a predictor of low QOL, in addition to clarifying how the intensity of depressive symptoms interacts with QOL and vice-versa. Fourth, the results cannot be generalized since most participants are medical, nursing and health students. Fifth, excluding gray research sources from our systematic review may resulted in loss of information on the subject. So, for future studies, we suggest to take into account the possibility to include a gray literature search as a step of the search strategy. Finally, the studies did not analyze important factors mediating in the relationship between QOL and mental health, such as socioeconomic level, stress, coping style, and personality ^112,130,131^.

The strengths of this study include the specific assessment of depression, to the detriment of a wide scope of mental health problems, which allows a particular analysis of its relationship with QOL. Results from the analysis of conflicts of interest and ethical approvals, which are often omitted from the assessments, are also presented here. A meta-analysis was conducted to provide a general estimate of the prevalence of depression among high school and university students. To the best of our knowledge, this is the first systematic review that summarizes the evidence on the relationship between depression and QOL among high school and university students, allowing us to clarify the gaps in the literature and propose recommendations for future research. In addition, this is the first study that intended to analyze academic consequences, such as academic performance, absenteeism, and school dropout. However, the studies included in this review did not analyze these aspects, which indicate a lack of research on the academic consequences, from the perspective of the relationship between depression and QOL.

New studies should be conducted considering the severity, duration, and patterns of depressive symptoms in high school and university students, to better understand the relationship between depression and QOL. Future research directions also include in-depth study on the relationship between depressive symptoms and specific dimensions of QOL, considering its domains and sub-domains, identification of sociodemographic variables and the influence of coping mechanisms on the relationship between depression and QOL, and longitudinal assessment of the relationship between depression and QOL among students. Health professionals and education professionals must better understand the different aspects of the life of students who are depressed, being able to determine its origin and the protection mechanisms that can be used in punctual interventions ^68,131^.

## Conclusion

Depression is associated with the QOL of students; however, the relationship between depression and QOL is not clear yet. There is a need to understand whether QOL can affect the nature, duration, and intensity of depressive symptoms and the real impact of depressive symptoms on different QOL domains. The consolidation of these findings is fundamental to a more effective and integrated orientation of public health and education policies, focusing on promoting mental health and improving the students’ QOL. The multidimensional aspect that refers to the students’ mental health and QOL should be considered from a multidisciplinary and global conception, with the participation of health professionals, education professionals and the family in social and instrumental support, thus contributing to students’ academic performance and success.

## Supplementary Information

Supplementary Information.

## Data Availability

Due to sensitive data, the data can be accessed upon request to the authors (michelevaladao2021@gmail.com (MSVF); matias.noll@ifgoiano.edu.br (MN)).
