# Supplementary material for: Relationship between depression and quality of life among students: a systematic review and meta-analysis
Source: Sci Rep. 2023 Apr 25;13:6715. doi: 10.1038/s41598-023-33584-3 (PMC10126541; doi:10.1038/s41598-023-33584-3)
Supplement: Supplementary file 1 — Supplementary Information. [file 41598_2023_33584_MOESM1_ESM.docx]

**Supplementary File 1**. Outcome Variables

| **Author/Year** | **Main result (Depression and QOL)** |
| --- | --- |
| Al-fayez and Ohaeri, 2011 | QOL^ⅰ^ is negatively correlated with depression (rho > -0.40, p < 0.05).  Depression is associated with worse QOL^ⅰ^ (p < 0.001). |
| Assana; Laohasiriwong;  Rangseekajee, 2017 | Absence of depression is associated with better QOL** (OR = 3.92; 95% CI: 2.94 − 5.23; p < 0.001). |
| Fernandes et al., 2022 | Depressive symptoms were associated with moderate (PR: 2.87, 95% CI: 1.68 − 4.89) and  low QoL (PR: 5.66; 95% CI: 3.48 − 9.19). |
| Gómez-Delgado et al., 2022 | HRQOL was significantly correlated with depression (p < 0.01), except in the dimensions financial resources and social support. The dimensions mood and emotions (r= - 0.060, p = 0.038), school environment (r= - 0.056, p = 0.011),  and social acceptance (r = - 0.040, p = 0.018), of HRQOL were identified as potential predictors of depression. |
| Ra and Cho, 2017 | Depression was negatively associated with HRQOL (β = - 0.599, p < 0.001). |
| Shin, Jeon and Cho, 2022 | HRQOL was significantly correlated with depression (r = -0.61, p <  0.01). |
| Stheneur et al., 2017 | The QOL was negatively related to the depression (r = - 0.5, p < 0.001). |
| Tekin, 2022 | QOL^i^ is negatively correlated with depression (r =- 0.621, p < 0.001).  Psychosocial QOL (r = - 0.675, p < 0.001).  Physical health QOL (r = - 0.311, p < 0.001). |
| Yang et al. 2022 | The somatization and depression subscales of SCL-90-R were significantly negatively associated with the SF-36 total scores (standardized partial regression coefficients β = - 0.170, β = - 0.372, respectively). |
| Albani et al., 2022 | Depression was predictive of QOL^i^.  Physical domain (β = - 0.923 (p = 0.003), Mental domain (β = - 1687 (p = 0.003). |
| Alvi et al., 2020 | QOL as the predictor variable depicted 11.4% variance in depression as outcome variable (F (1.198) = 26.61, p < 0.001).  QOL was negatively related to depression (β = - 0.12, p < 0.001). |
| Angkurawaranon et al., 2016 | Depression is associated with low HRQL^i^ in the mental domains (β = - 36.31, 95% CI: -41.82 − -30.78, p < 0.01) and physical (β = - 18.14 95% CI: -24.00 – 12.28, p < 0.01). |
| Aqeel et al., 2020 | T0: There was a significant decline in quality of life of students with different levels of depression, as normal (M = 25.96, SD = 0.20), mild (M = 26.00, SD = 0.00, n = 4), moderate (M = 26.00, SD = 0.00), and severe (M = 25.85, SD = 0.37).  T1: There was improvement in the quality of life with different levels of depression such as normal (M = 95.54, SD = 19.28), mild (M = 89.16, SD = 10.98), moderate (M = 83.60, SD = 5.94), and severe (M = 91.60, SD = 17.05).  T2: participants illustrated more slight improvement in quality of life with higher level of depression such as normal (M = 105.58, SD = 11.38), mild (M = 100.00, SD = 7.00), moderate (M = 90.55, SD = 3.29), and severe (M = 88.50, SD = 13.22). |
| Armoon et al. 2019 | Depression is not correlated with QOL** (OR = 2.91; 95% CI: 0.50 − 16.89, p = 0.23). |
| Blebil et al., 2021 | Significant negative moderate correlation was found between depression versus quality of life (r = - 0.59, p = 0.01). |
| Borges et al., 2020 | Students of the 1st and 3rd semesters: Depressive symptoms is negatively correlated poorer psychological QOL (β*=* -2.82, p < 0.0001).  Students of the 8st and 12rd semesters: Depressive symptoms is negatively correlated poorer psychological QOL (β *=* -3.04, p < 0.0001). |
| Burger et al., 2016 | There were correlations between depression and mental QOL in all semesters (r = - 0.70, p < 0.01). |
| Cleofas, 2020 | QOL** is negatively correlated with depression (r = - 0.320; p = 0.000). |
| Gan and Rue, 2019 | Depressive symptoms are associated with lower general QOL (χ^2^ =3.41, p < 0.001), except with the general health domain.  Significant symptoms of depression are not associated with the general health (χ^2^ = 4.0, p = 0.9) and social domains (χ^2^ = 57.6, p = 0.2). |
| Ghassab‑Abdollahi, et al., 2020 | There were indirect significant correlations between total score of QOL and its all subdomains with Beck depression score adjusted for covariates (Total QOL: β = - 0.85 (p < 0.001).  Depression negatively predicted QoL (β = - 0.8, p < 0.001). |
| Jenkins et al., 2020 | Depression is correlated with QOL^i^ (rho ranged from - 0.197 to - 0.768, p < 0.01).  Depression is not correlated with physical functioning (β *=* - 0.92, p= 0.247) and pain (β = - 0.068, p = 0.375). |
| Karuniawaiti et al., 2022 | Depression was significantly correlated with the QOL^i^.  Physical domain (r=−0.393, p<0.001), Psychological domain (r = - 0.161, p < 0.001) and Social relationship (r = - 0.400, p < 0.001). Environmental domain (r = - 0.040, p = 0.325). |
| Li et al., 2020 | Depression is positively associated with low QOL^i^ (p < 0.001). |
| Marković et al., 2022 | Correlation between the quality of life and depression (r = 0.613, p < 0.001). |
| Miguel et al., 2021 | Depressive symptoms are predictors QOL (QOL in students with depressive symptoms is lower, 6.96 (CI: 6.85, 7.06) vs. 6.02 (CI: 5.90, 6.13), p < 0.001). |
| Moutinho et al., 2019 | Psychological (β = - 0.24, p < 0.001), social (β = - 0.236), p < 0.001), and environmental QOL (β = - 0.151, p = 0.018) in wave 4 (T1) were negatively associated with depression in wave 1 (T4). |
| Pagnin and Queiroz, 2015 | Increased depressive symptoms negatively impact the psychological dimensions (β=−0.64, 95% CI: −0.84 to −0.45, p<0.001), social (β = - 0.51, 95% CI: - 0.85 to - 0.17, p < 0.001), and environmental dimensions (β = - 0.38, 95% CI: - 0.61 to - 0.15, p = 0.001) of QOL. |
| Pecmezovic et al., 2011 | HRQL^i^ is negatively correlated with depression (ranged from 0.304 to - 0.618, p = 0.001). |
| Pillay, Ramlall and Burns, 2016 | Negative correlation between depressive symptoms and QOL^i^ (ρ = - 0.483, p = 0.000). |
| Racic et al., 2017 | There is no significant association between depression and HRQL** (OR = -0.36, 95% CI: -0.88–0.009, p = 0.115). |
| Ratnani et al., 2017 | Students with depression are likely to have low QOL^i^ (p < 0.0001). |
| Singh et al., 2016 | Depression negatively impacts the physical (r = - 0.544, p = 0.0001), psychological (r = - 0.521, p = 0.0001), and environmental (r = - 0.382, p = 0.006) domains of QOL in medical students.  Depression negatively impacts the physical (r = - 0.512, p=0.0001) and social (r= - 0.321, p = 0.023) domains of QOL in engineering students.  Depression does not negatively impact the QOL of art students. |
| Solanki et al., 2021 | Depression is correlated with QOL^i^.  Physical domain (OR = 0.97, 95% CI: - 0.95 − 0.99, p = 0.008), Psychological domain (OR = 0.95, 95% CI: - 0.93 − 0.97, p<0.001) and Social relationship (OR = 0.97, 95% CI: -0.95 − 0.99, p = 0.001). Environmental domain, NR. |
| Souza et al., 2012 | Moderate symptoms of depression negatively affect the mental (ES=0.8, p=0.00) and physical components (ES = 0.7, p = 0.00) of HRQOL (p = 0.00). |
| Tejoyuwono, Nugraha and Fahdi, 2021 | Depression influenced QOL in physical aspects health domain (χ^2^ = 20.598, p < 0.001), psychological health domain (χ^2^ = 24.963, p<0.001) and social relations domain (χ^2^ = 29.808, p<0.001). |
| Wen et al., 2022 | QOL and depression were also highly correlated (r= - 0.601, p < 0.001).  QOL negatively predicted depression (β = - 0.197, p < 0.001). |
| CI, Confidence interval; ES, Effect size; F, Variance test; M, median; OR, Odds ratios; SD, standard deviation; T, reference for the data collection period in the longitudinal study.  **, does not present analysis of different domains of QOL/HRQOL.  ^ⅰ^, the different QOL / HRQOL domains were analyzed separately.  β, regression coefficient; ⅰ, the different QOL / HRQOL domains were analyzed separately.  ρ, correlation coefficient; p, p-value - tested with a significance level of 0.05.  r, Pearson's correlation coefficient; rho, Spearman's correlation coefficient; χ^2^, chi-squared test. | |
